# The predictive value of FAH model for the occurrence of colorectal cancer

**DOI:** 10.3389/fmed.2025.1512173

**Published:** 2025-03-07

**Authors:** Zhixuan Ma, Qing Wu, Qingming Wu

**Affiliations:** ^1^School of Public Health, Wuhan University of Science and Technology, Wuhan, China; ^2^Department of Gastroenterology, Ziyang People's Hospital, Ziyang, China; ^3^Department of Critical Care Medicine, Ziyang People's Hospital, Ziyang, China

**Keywords:** colorectal cancer, fatty liver, carotid atherosclerosis, high-density lipoprotein cholesterol, intestinal dysbiosis

## Abstract

**Background:**

Fatty liver is characterized by hepatic steatosis and is associated with dyslipidemia and insulin resistance. Carotid atherosclerosis, characterized by plaque formation, may be related to increased lipid deposition. High-density lipoprotein cholesterol (HDL-C) plays a role in reverse cholesterol transport. Colorectal cancer (CRC) is significantly associated with lipid metabolism-related diseases. However, there is a paucity of research on the relationship between lipid metabolism disorders and CRC.

**Objective:**

To determine whether fatty liver (F), carotid atherosclerosis (A), and HDL-C (H) models (FAH) have predictive value for the occurrence of CRC and can be used for CRC screening.

**Methods:**

A case–control study was conducted on 166 patients with CRC and 448 patients who underwent physical examinations at Ziyang People’s Hospital between September 2018 and August 2023. A 1:3 individual matching strategy was used to establish the independent risk factors for CRC using univariate and multivariate analyses. A model was constructed based on independent risk factors, and its accuracy and sensitivity were verified. The discriminative ability, calibration, and clinical utility of the predictive model were evaluated using the Receiver Operating Characteristic curve, bootstrap resampling method, the Hosmer–Lemeshow goodness-of-fit test, and Decision Curve Analysis (DCA).

**Results:**

Fatty liver (F), carotid atherosclerosis (A), HDL-C (H), and intestinal dysbiosis (D) were identified as independent risk factors for CRC. The odds ratios were 2.885, 11.452, 24.659, and 22.445, respectively, *p* < 0.001. Based on these results, an FAH prediction model was established. The Horser–Lemeshow test for the FAH prediction model yielded *p* = 0.710. The cut-off value was 0.275, with the area under the curve of 0.902 (95% Confidence Interval: 0.875–0.929), *p* < 0.001. The sensitivity was 86.7%, and the specificity was 78.1%. A nomogram was created, and the internal calibration chart showed that the calibration curve closely aligned with the standard curve, indicating good discrimination and predictive ability of the model. DCA demonstrated that the model had a favorable clinical net benefit.

**Conclusion:**

The FAH model has predictive value for CRC occurrence owing to its noninvasive nature and easy availability of data, making it worthy of further clinical research.

## 1 Introduction

Colorectal cancer (CRC) is a malignant tumor originating from the mucosal epithelium of the colon and rectum. Globally, CRC ranks first in terms of number of new cases in the United States and China (1). In China, CRC is the leading cause of cancer-related mortality (2). Early detection of CRC is important to improve patient survival and reduce mortality rates. Numerous recent domestic and international studies have indicated that metabolic syndrome (MS), nonalcoholic fatty liver disease (NAFLD), atherosclerosis, and other metabolic disorders are closely associated with an increased CRC incidence (3–6). MS includes dyslipidemia, NAFLD, diabetes mellitus, and obesity (7). Current early diagnostic methods for CRC, including blood and fecal tumor marker detection (8), have limited sensitivity and specificity. Colonoscopy is considered the gold standard for CRC screening (9); however, its invasive nature and low patient compliance limit its application in large-scale screening. NAFLD is a type of fatty liver disease, and carotid atherosclerosis is a manifestation of carotid artery atherosclerosis. Based on the metabolic characteristics of these diseases, we speculated that fatty liver, carotid atherosclerosis, and high-density lipoprotein cholesterol (HDL-C) levels may be correlated with the occurrence and progression of CRC. As these indicators can be conveniently and rapidly obtained through ultrasound and blood tests, their potential application in CRC screening deserves further research. Gut microbiota dysbiosis is considered a significant factor affecting CRC development (10). To further explore the correlation between these metabolic indicators and CRC, we selected patients from Ziyang People’s Hospital as participants for our study with the aim of assessing the potential value of fatty liver, carotid atherosclerosis, HDL-C, and gut microbiota dysbiosis in CRC screening.

## 2 Materials and methods

### 2.1 Study participants

This study included patients who visited Ziyang People’s Hospital between September 1, 2018, and August 31, 2023, as clinical research participants.

The inclusion criteria were as follows: (1) Age over 18 years. (2) Availability of results for liver ultrasound, carotid vascular ultrasound, blood lipid levels, fecal rod-to-sphere ratio, and colonoscopy. (3) Diagnosis of fatty liver based on four known criteria (11): liver-kidney echo contrast, liver brightness, deep attenuation, and vessel blurring. Fatty liver is classified into three groups according to severity. Mild fatty liver is defined as a slight increase in liver echogenicity. Moderate fatty liver is defined as mild visual impairment of the hepatic vessels and diaphragm, along with increased liver echogenicity. Severe fatty liver is defined as a significant increase in liver echogenicity, poor penetration in the posterior segment of the right lobe, and poor or absent visualization of hepatic vessels and diaphragm. (4) Diagnosis of carotid atherosclerosis conformed to the Chinese Guidelines for Vascular Ultrasound Examination in Stroke (12): Ultrasound was used to assess carotid plaques and carotid intima-media thickness (CIMT). Certified sonographers manually traced a 10 mm segment of the carotid artery’s intima-media interface using two-dimensional grayscale ultrasound images at the end of diastole and measured the CIMT at three sites: the distal common carotid artery, carotid bulb, and proximal internal carotid artery. CIMT was considered abnormal if the maximum value at the three measurement points was ≥1.0 mm. The presence of plaque was defined as CIMT >1.5 mm, a focal structure encroaching >0.5 mm into the arterial lumen, or > 50% of the adjacent CIMT. (5) CRC must meet the pathological diagnostic criteria (13). (6) The diagnostic criteria for gut microbiota dysbiosis based on the Chinese Expert Consensus on Clinical Application of Microecological Agents (2020 Edition) (14): medical history indicating a primary disease causing an imbalance in the gut microbiota; clinical manifestations of gut microbiota imbalance, such as diarrhea, bloating, abdominal pain, and abdominal discomfort; laboratory evidence of gut microbiota imbalance: fecal smear examination showing a cocci/bacilli ratio (the reference value for adults is 1:3).

The exclusion criteria were as follows: (1) Incomplete medical history. (2) History of previous malignant tumors and inflammatory bowel diseases, such as ulcerative colitis or Crohn’s disease. (3) Previous colectomy for any reason. (4) Incomplete colonoscopy results. This study was approved by the Ethics Committee of Ziyang People’s Hospital (Ethics Committee Approval No. 20230901), and informed consent was obtained from all patients.

### 2.2 Research methods

According to the research design, individual matching was conducted at a 1:3 ratio, followed by sex matching. A total of 614 patients were included in the study, with 166 patients with CRC in the study group and 448 patients without CRC in the control group.

### 2.3 Clinical data collection

Data on age, sex, height, weight, smoking, alcohol consumption, aspirin use history, family history of CRC (first-degree relatives), history of hypertension, and diabetes were collected. Body mass index (BMI) was calculated as BMI = body mass (kg) / height^2^ (m^2^).

### 2.4 Platelet count, serum biochemistry, lipid, and carcinoembryonic antigen testing

After fasting for 12 h, peripheral venous blood was collected to test for platelet count (Plt), alanine transaminase (ALT), aspartate aminotransferase (AST), gamma-glutamyltransferase (GGT), total bilirubin (TBil), indirect bilirubin (IBiL), direct bilirubin (DBiL), total bile acid (TBA), total cholesterol (TC), HDL-C, low-density lipoprotein cholesterol (LDL-C), triglycerides (TG), creatinine (Cre), and carcinoembryonic antigen (CEA).

### 2.5 Fecal rod-to-sphere ratio

Fecal rod-to-sphere ratio analysis was performed using an Olympus CX22LED microscope, manufactured in Japan.

### 2.6 Liver and carotid vascular ultrasound examination

After fasting for 8–12 h, in the supine position, a Siemens ACUSON S2000 color ultrasound diagnostic system was used, and liver and carotid vascular ultrasound examinations were uniformly conducted by sonographers.

### 2.7 Colonoscopy

All patients completed a full colonoscopy.

### 2.8 Pathology report materials

Pathologists wrote complete pathology reports based on the characteristics of the CRC tissue sections under a microscope, which were reviewed by senior physicians.

### 2.9 Statistical analysis

Statistical analyses were performed using SPSS 19.0 and R 4.4.1 software. Normally distributed quantitative data were expressed as mean ± standard deviation (x ± s) and analyzed using *t*-tests, while non-normally distributed quantitative data were expressed as median and interquartile range M (Q_1_, Q_3_), and comparisons between groups were made using the Mann–Whitney U test. Categorical data were expressed as numbers (%). Chi-square tests were used for unordered categorical data, and Mann–Whitney U tests were used for ordered categorical data. Pearson and Spearman correlation tests were used for correlation analysis of normally and non-normally distributed data, respectively. Binary Logistic regression analysis was used to identify independent risk factors affecting CRC occurrence and progression, and a predictive model formula was constructed. R language was used to construct the nomogram models. The area under the receiver operating characteristic (ROC) curve (AUC) was calculated to assess the predictive ability of the model. Internal validation of the model was performed using the bootstrap resampling method, with the construction of bootstrap-AUC and calibration curves. A *p*-value of less than 0.05 was considered statistically significant.

## 3 Results

### 3.1 Clinical characteristics of CRC and control groups

In this study, no statistically significant differences were observed between the CRC group and the control group in terms of gender, family history of CRC, heart failure, chronic kidney disease, cerebral infarction, fatty liver grade, AST, DBiL, GGT, Cre, TG, and LDL-C (*p* > 0.05). However, age, BMI, smoking, alcohol consumption, aspirin use, intestinal polyps, diabetes, hypertension, carotid atherosclerosis, chronic obstructive pulmonary disease (COPD), gut microbiota dysbiosis, fatty liver, Plt, ALT, AST/ALT ratio, TBil, IBiL, TBA, CEA, TC, and HDL-C showed statistically significant differences between the CRC group and control group (*p* < 0.05) (Table 1).

**Table 1:** Clinical characteristics of CRC and control groups.

Clinical characteristics	CRC group (*n* = 166)	Non-CRC group (*n* = 448)	*χ2/Z*	*p* value
Gender, *n* (%)				
Male	105 (63.3)	292 (65.2)	0.197^a^	0.658
Female	61 (36.7)	156 (34.8)		
Age [years, M (Q_1_, Q_3_)]	70 (61 ~ 76)	61 (53 ~ 70)	−6.230^b^	<0.001^*^
BMI (Kg/m^2^)	23.3 (20.9 ~ 25.3)	24.0 (22.5 ~ 25.9)	−2.887^b^	0.004^*^
Smoking, *n* (%)	68 (41.0)	105 (23.4)	−4.472^b^	<0.001^*^
Alcohol consumption, *n* (%)	60 (36.1)	96 (21.4)	−3.936^b^	<0.001^*^
Family history of CRC, *n* (%)	5 (3.0)	4 (0.9)	2.442^a^	0.118
Aspirin use, *n* (%)	12 (7.2)	9 (2.0)	−2.877^b^	0.004^*^
Intestinal polyps, *n* (%)	97 (58.4)	337 (75.2)	16.477^a^	<0.001^*^
Diabetes, *n* (%)	31 (18.7)	40 (9.0)	−3.141^b^	0.002^*^
Hypertension, *n* (%)	56 (33.7)	73 (16.3)	−4.270^b^	<0.001^*^
Heart failure, *n* (%)	2 (1.2)	0 (0.0)	–	0.073
Chronic kidney disease, *n* (%)	3 (1.8)	2 (0.5)	1.348^a^	0.246
Cerebral infarction, *n* (%)	27 (16.3)	51 (11.4)	2.602^a^	0.107
Carotid atherosclerosis, *n* (%)	98 (59.0)	61 (13.6)	130.209^a^	<0.001^*^
COPD, *n* (%)	16 (9.6)	16 (3.6)	−3.760^b^	<0.001^*^
Gut microbiota dysbiosis, *n* (%)	23 (13.8)	3 (0.6)	−7.200^b^	<0.001^*^
Fatty liver, *n* (%)	76 (45.8)	161 (36.0)	4.954^a^	0.026^*^
Fatty liver absent, *n* (%)	90 (54.2)	287 (64.1)	–	–
Mild fatty liver, *n* (%)	65 (39.2)	114 (25.5)	–	–
Moderate fatty liver, *n* (%)	11 (6.6)	46 (10.3)	–	–
Severe fatty liver, *n* (%)	0 (0.0)	1 (0.2)	–	–
Fatty liver grading	–	–	−1.637^b^	0.102
Platelet count (×10^9^/L)	216.5 (173.0 ~ 272.0)	186.5 (147.0 ~ 226.0)	−5.018^b^	<0.001^*^
ALT (U/L)	13.0 (8.5 ~ 20.3)	18.0 (12.9 ~ 28.1)	−5.683^b^	<0.001^*^
AST (U/L)	25.6 (20.4 ~ 32.5)	26.7 (21.5 ~ 33.4)	−1.499^b^	0.134
AST/ALT	1.80 (1.3 ~ 2.7)	1.4 (1.0 ~ 2.0)	−5.698^b^	<0.001^*^
Total bilirubin (umol/L)	12.0 (9.0 ~ 15.5)	13.3 (10.3 ~ 16.8)	−2.835^b^	0.005^*^
Direct bilirubin (umol/L)	4.1 (3.2 ~ 5.2)	4.2 (3.3 ~ 5.2)	−0.323^b^	0.747
Indirect bilirubin (umol/L)	7.6 (5.6 ~ 10.5)	9.0 (6.8 ~ 11.8)	−3.674^b^	<0.001^*^
GGT (U/L)	19.8 (14.3 ~ 34.1)	21.4 (14.8 ~ 33.5)	−0.587^b^	0.557
Total bile acids (umol/L)	4.0 (2.2 ~ 6.5)	3.3 (1.6 ~ 5.8)	−2.682^b^	0.007^*^
Serum creatinine (umol/L)	64.8 (54.1 ~ 78.5)	64.4 (52.6 ~ 76.4)	−1.243^b^	0.214
Carcinoembryonic antigen (ng/mL)	3.9 (2.1 ~ 10.9)	2.0 (1.2 ~ 2.4)	−10.559^b^	<0.001^*^
Total cholesterol (mmol/L)	4.2 (4.1 ~ 4.7)	4.6 (4.1 ~ 5.4)	−4.665^b^	<0.001^*^
Triglycerides (mmol/L)	1.28 (0.96 ~ 2.34)	1.28 (0.86 ~ 2.18)	−0.399^b^	0.690
HDL-C (mmol/L)	3.11 (3.11 ~ 3.11)	1.24 (1.02 ~ 1.53)	−13.552^b^	<0.001^*^
LDL-C (mmol/L)	2.74 (2.24 ~ 3.74)	2.52 (2.01 ~ 3.20)	−0.721^b^	0.471

a is χ^2^; b represents the z-value; *: *p* < 0.05; CRC, colorectal cancer; BMI, body mass index; COPD, chronic obstructive pulmonary disease; ALT, alanine aminotransferase; AST, aspartate aminotransferase; AST/ALT, ratio of aspartate aminotransferase to alanine aminotransferase; ALP, alkaline phosphatase; GGT, gamma-glutamyltransferase; HDL-C, high-density lipoprotein cholesterol; LDL-C, low-density lipoprotein cholesterol.

### 3.2 Analysis of CRC risk factors

Correlation analysis of variables with *p* < 0.05 revealed that ALT, TBil, IBiL, TC, BMI, and intestinal polyps were negatively correlated with CRC, with correlation coefficients of −0.161, −0.097, −0.130, −1.71, −0.129, and − 0.164, respectively. Age, diabetes mellitus, hypertension, carotid atherosclerosis, fatty liver, COPD, smoking, alcohol consumption, aspirin use, gut microbiota dysbiosis, Plt, AST/ALT ratio, TBA, HDL-C, and CEA were positively correlated with CRC. Multivariate regression analysis, which incorporated the positively correlated variables, identified age, diabetes mellitus, carotid atherosclerosis, fatty liver, COPD, gut microbiota dysbiosis, Plt, HDL-C, and CEA as risk factors for CRC (Table 2). The diagnoses of fatty liver, carotid atherosclerosis, HDL-C, and intestinal microbiota dysbiosis were based on variance inflation factor (VIF) less than 5, indicating no multicollinearity among the variables. The inclusion of variables was determined by considering the current research, clinical theory, data collection feasibility, cost-effectiveness, and the efficacy of computation and prediction. Binary Logistic regression analysis demonstrated that fatty liver, carotid atherosclerosis, HDL-C, and gut microbiota dysbiosis were independent risk factors for CRC, with odds ratios (OR) of 2.885, 11.452, 24.659, and 22.445, respectively (*p* < 0.001, Table 3). By streamlining variable selection, the aim was to develop a predictive model that was both cost-effective and clinically practical, providing an accurate tool for the risk assessment of CRC.

**Table 2:** Analysis of colorectal cancer risk factors.

Parameters	Correlation analysis			Multivariable logistic regression analysis		
	Correlation coefficient	*p* value		OR	95% confidence interval	*p* value
Age	0.247	<0.001^*^		1.061	1.026 ~ 1.079	<0.001^*^
Diabetes mellitus	0.127	0.002^*^		7.032	3.317 ~ 14.905	<0.001^*^
Hypertension	0.172	<0.001^*^		0.959	0.448 ~ 2.056	0.915
Carotid atherosclerosis	0.461	<0.001^*^		6.879	3.420 ~ 13.836	<0.001^*^
Fatty liver	0.090	0.026^*^		3.118	1.606 ~ 6.052	0.001^*^
COPD	0.152	<0.001^*^		4.659	1.364 ~ 15.913	0.014^*^
Smoking	0.181	<0.001^*^		2.501	0.942 ~ 6.636	0.066
Alcohol consumption	0.150	<0.001^*^		1.363	0.497 ~ 3.738	0.548
Aspirin use	0.116	0.004^*^		0.870	0.155 ~ 4.875	0.874
Gut microbiota dysbiosis	0.291	<0.001^*^		19.887	2.752 ~ 143.744	0.003^*^
Platelet count	0.241	<0.001^*^		1.008	1.004 ~ 1.012	<0.001^*^
AST/ALT	0.166	<0.001^*^		1.113	0.976 ~ 1.268	0.111
Total bile acids	0.089	0.027^*^		1.012	0.959 ~ 1.068	0.669
HDL-C	0.741	0.001^*^		29.002	14.158 ~ 59.418	<0.001^*^
Carcinoembryonic antigen	0.426	<0.001^*^		1.081	1.035 ~ 1.129	<0.001^*^

OR, odds ratio; *: *p* < 0.05; COPD, chronic obstructive pulmonary disease; AST/ALT, ratio of aspartate aminotransferase to alanine aminotransferase; HDL-C, high-density lipoprotein cholesterol.

**Table 3:** Binary logistic analysis of independent risk factors for colorectal cancer.

Risk factors	*B*	SE	Wald	*p* value	*OR*	OR95%CI	
						Lower limit	Upper limit
Fatty Liver	1.059	0.275	14.878	<0.001	2.885	1.684	4.942
Carotid atherosclerosis	2.438	0.291	70.417	<0.001	11.452	6.480	20.239
HDL-C	3.205	0.294	118.790	<0.001	24.659	13.857	43.882
Dysbiosis of the gut microbiota	3.111	0.762	16.649	<0.001	22.445	5.036	100.026

S, regression coefficient; SE, standard error; Wald, statistical test; OR, odds ratio; CI, confidence interval; HDL-C, high-density lipoprotein cholesterol.

### 3.3 Establishment of the FAH model

The optimal cutoff value for HDL-C was 1.615 mmol/L, as determined by ROC analysis. Based on the results of the binary logistic regression analysis, predictive models with different combinations of four risk factors—fatty liver (F), carotid atherosclerosis (A), high-density lipoprotein cholesterol (H), and gut microbiota dysbiosis (D)—were constructed. Cutoff values, AUC, sensitivity, specificity, and 95% confidence intervals (CI) of the models were calculated (Table 4). After a multidimensional assessment of model stability, AUC, ease of operation, and cost-effectiveness, both the FAHD and FAH models demonstrated high AUC, sensitivity, specificity, and 95% CI. Considering that the FAH model is more economical, convenient, and rapid than the FAHD model and given that many primary hospitals in China have not yet implemented fecal microbiota ratio testing, the optimal FAH model, which includes fatty liver, carotid atherosclerosis, and HDL-C, was ultimately selected. The Hosmer–Lemeshow goodness-of-fit test for the FAH model yielded a *p*-value of 0.710, indicating a good model fit. The formula for the model is: CRC risk = −3.803 + 1.082 × fatty liver +2.443 × carotid atherosclerosis +3.165 × HDL-C (Table 5).

**Table 4:** Efficacy analysis of various models in predicting colorectal cancer.

Model	Cut-off value	AUC	Sensitivity	Specificity	95%CI	*p* value
FAHD	0.311	0.913	0.892	0.780	0.887 ~ 0.939	<0.001
FAH	0.275	0.902	0.867	0.781	0.875 ~ 0.929	<0.001
FA	0.381	0.760	0.590	0.864	0.714 ~ 0.805	<0.001
AH	0.166	0.884	0.934	0.708	0.853 ~ 0.914	<0.001
AHD	0.146	0.898	0.946	0.703	0.870 ~ 0.926	<0.001

CRC, colorectal cancer; AUC, area under the curve; CI, confidence interval; FAHD, Fatty Liver + Carotid Atherosclerosis + High-Density Lipoprotein Cholesterol + Gut Microbiota Dysbiosis; FAH, Fatty Liver + Carotid Atherosclerosis + High-Density Lipoprotein Cholesterol; FA, Fatty Liver + Carotid Atherosclerosis; AH, Carotid Atherosclerosis + High-Density Lipoprotein Cholesterol; AHD, Carotid Atherosclerosis + High-Density Lipoprotein Cholesterol + Gut Microbiota Dysbiosis.

**Table 5:** Variables in the FAH model equation.

Risk factors	*B*	SE	Wald	*p* value	OR	OR95%CI	
						Lower limit	Upper limit
Fatty liver	1.082	0.265	16.657	<0.001	2.951	1.755	4.963
Carotid atherosclerosis	2.443	0.281	75.434	<0.001	11.513	6.633	19.983
HDL-C	3.165	0.282	126.093	<0.001	23.683	13.631	41.146
Constants	−3.803	0.303	157.781	<0.001	0.022	–	–

S, regression coefficient; SE, standard error; Wald, Wald test statistic; OR, odds ratio; CI, confidence interval; HDL-C, high-density lipoprotein cholesterol.

**Figure 1:**
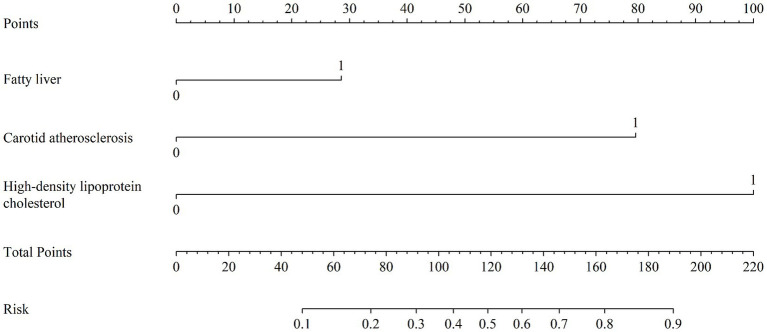
Nomogram model for predicting the risk of colorectal cancer.

The nomogram model was plotted using R 4.4.1 software (Figure 1). For example, a patient with fatty liver, carotid atherosclerosis, and an HDL-C level above 1.615 mmol/L would have a total score indicating a 94% risk of CRC occurrence, according to the nomogram model. This suggests that the higher the total score of the nomogram model, the higher is the risk of CRC in the patient.

### 3.4 Clinical efficacy and validation of the FAH model

**Figure 2:**
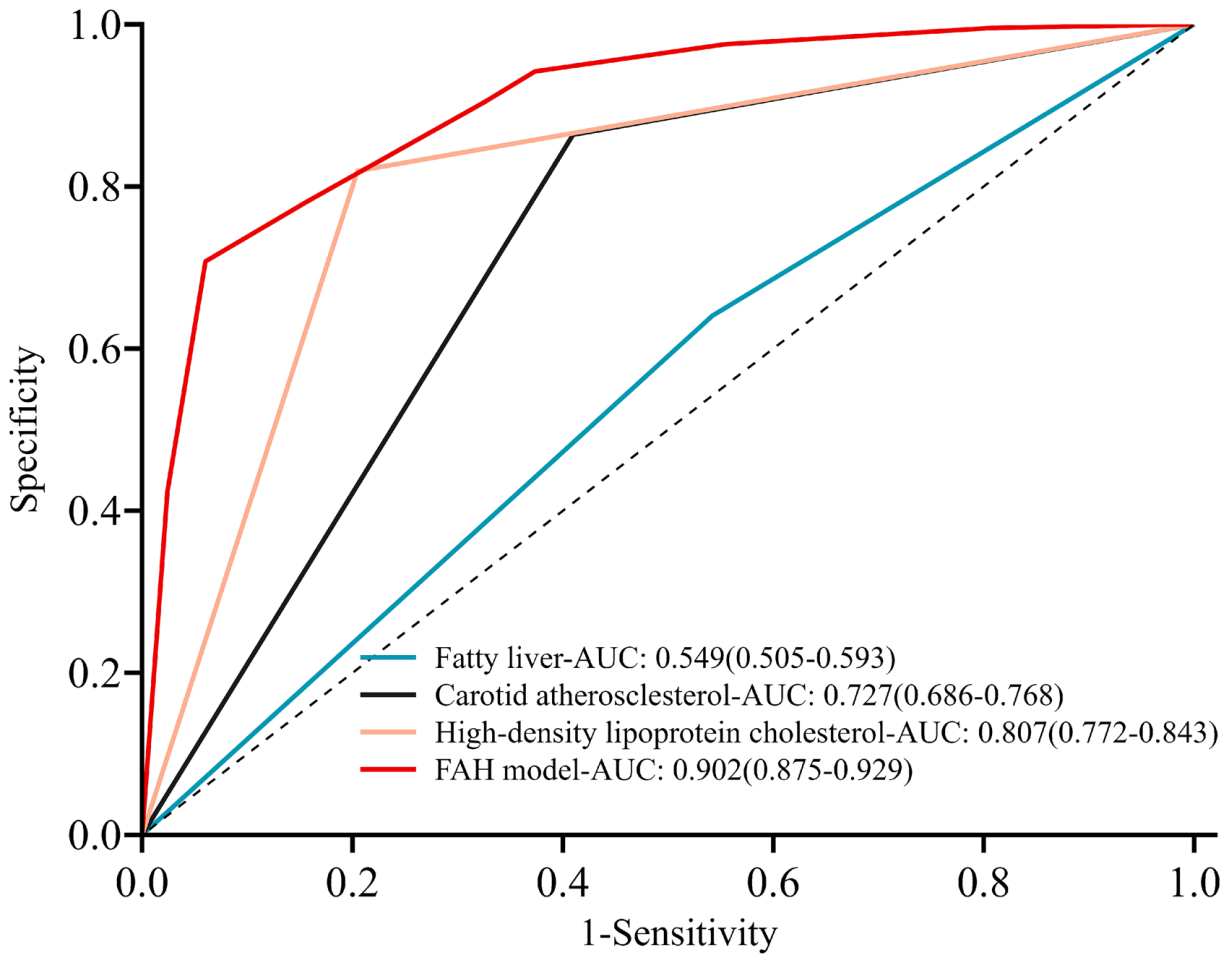
Clinical efficacy of three risk factors and the FAH model in predicting colorectal cancer.

**Figure 3:**
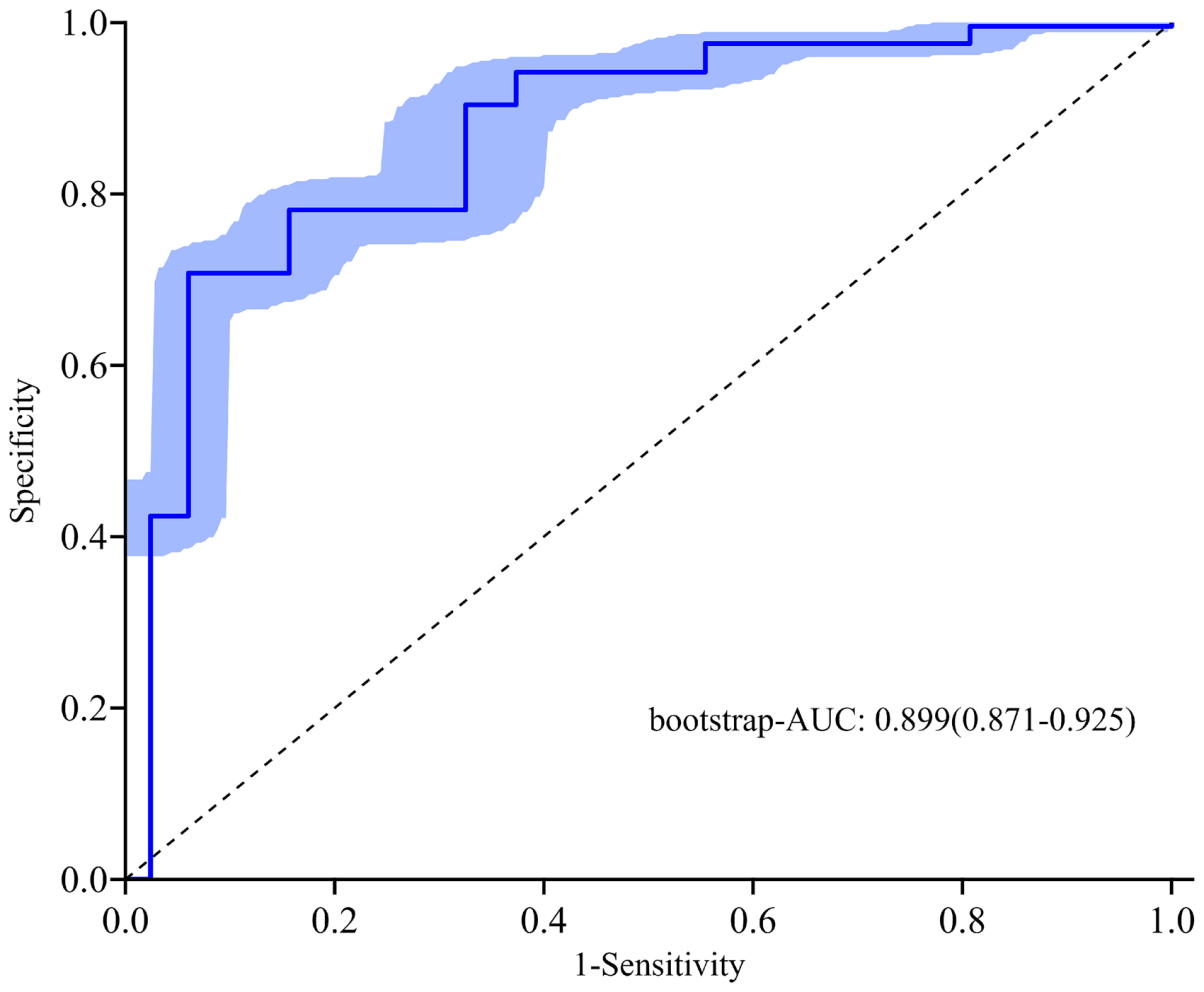
Bootstrap-AUC of the FAH model.

**Figure 4:**
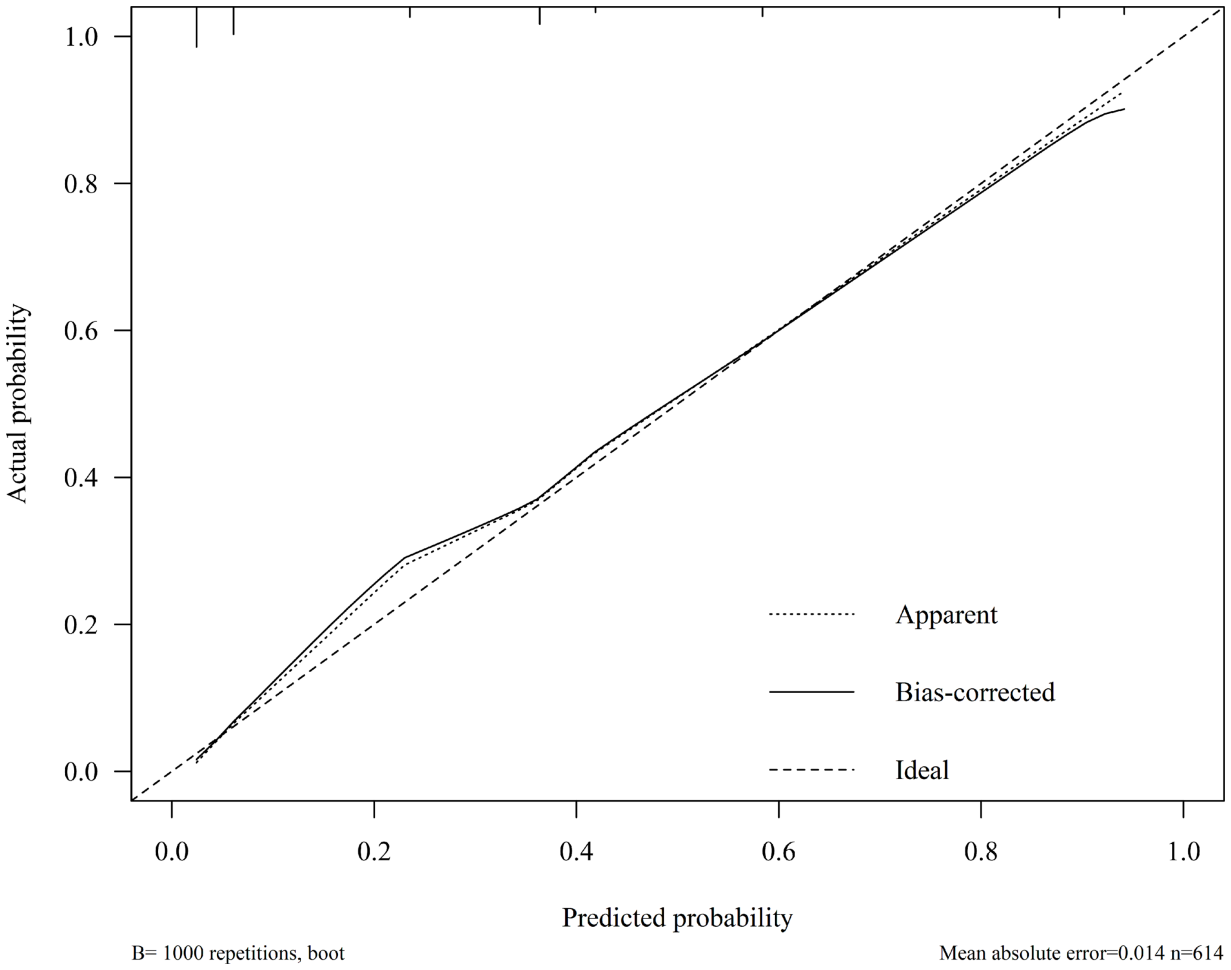
Calibration curve of the FAH model.

**Figure 5:**
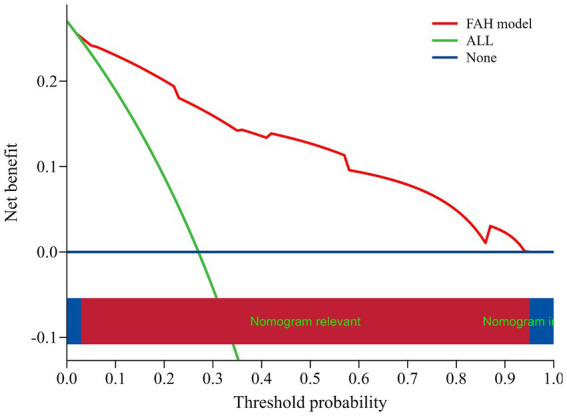
DCA curve of the FAH model.

ROC curves were plotted for the FAH model and the individual components of fatty liver, carotid atherosclerosis, and HDL-C to obtain their respective AUCs. The AUC for the FAH model in predicting CRC was 0.902 (95% CI: 0.875–0.929), with a sensitivity of 86.7% and a specificity of 78.1% (*p* < 0.001). The AUCs for predicting CRC with fatty liver disease, carotid atherosclerosis, and HDL-C levels were 0.549, 0.727, and 0.807, respectively (Figure 2). The predictive ability of each individual risk factor for CRC was lower than that of the FAH model, indicating the model’s strong discriminative power. Internal validation of the model was performed using the bootstrap resampling method with 1,000 repetitions. The adjusted bootstrap-AUC was 0.899 (95% CI: 0.871–0.925), *p* < 0.001 (Figure 3). The calibration curve after internal validation revealed that the bias-corrected calibration curve of the model was close to the ideal curve, indicating good predictive stability and consistency (Figure 4). Decision Curve Analysis demonstrated that the model had a favorable clinical net benefit (Figure 5).

## 4 Discussion

### 4.1 CRC screening methods

CRC remains the second leading cause of cancer-related deaths (15), with a 5-year survival rate of up to 90.6% for early-stage CRC, compared to only 14.7% for late-stage CRC (16). Despite the effectiveness of clinical screening in reducing CRC incidence and mortality, data published by the National Cancer Center highlight persistent high risk and fatality rates across different age groups (17), indicating the need for continued efforts to develop CRC screening methods. Current screening methods, such as colonoscopy, are highly invasive and have low acceptance. Serum and fecal tumor marker screenings, although easy to perform, have insufficient specificity, limiting their application in large-scale screening. Although it is highly sensitive and specific, fecal DNA screening is costly and difficult to promote routinely. Moreover, comprehensive screening strategies, although theoretically comprehensive, are still controversial regarding their long-term efficacy and practical feasibility. Therefore, CRC screening faces many challenges in terms of method selection, patient compliance, risk of overdiagnosis, personalized screening strategies, application of emerging technologies, and resource allocation. Further research and improvements are needed to enhance the effectiveness and feasibility of CRC screening.

### 4.2 Effectiveness of the FAH model in predicting CRC

This study constructed an FAH prediction model using binary logistic regression analysis. The risk prediction nomogram model can intuitively display the impact of each risk factor on the occurrence of CRC in patients, providing clinical guidance for medical staff to perform colonoscopies for CRC screening. The model was tested using the Hosmer–Lemeshow goodness-of-fit test to enhance the scientific and rigorous nature of the modeling process. An AUC above 0.9 indicates good prediction performance (18), and the AUC of the model in this study was 0.902 with a cut-off value of 0.275, sensitivity of 86.7%, specificity of 78.1%, and a 95% CI of 0.875–0.929 (*p* < 0.001). This suggests that the model has a diagnostic value for predicting CRC. Internal validation of the model showed a sensitivity of 94%, specificity of 70.8%, and accuracy of 77%, indicating that the prediction model had good practical predictive ability.

### 4.3 Theoretical basis and risk factor analysis of FAH model

Fatty liver, carotid atherosclerosis, and HDL-C are closely related to lipid metabolism and play key roles in the occurrence and development of CRC. Dyslipidemia provides tumor cells with abundant energy, nutrients, and redox requirements, supporting their malignant growth and metastasis (19). A study based on circular RNA microarray revealed that circCAPRIN1 can promote CRC progression, elucidating the molecular mechanism by which circular RNA promote tumor progression through dysregulated lipid metabolism (20). Dyslipidemia is a hallmark of cancer (21). Multiple enzymes, proteins, and transcription factors participate in the reprogramming of CRC lipid metabolism. Their abnormal expression promotes lipid synthesis and droplet accumulation through various mechanisms, thereby affecting the growth, proliferation, and metastasis of CRC cells.

This study found that fatty liver and carotid atherosclerosis were independent risk factors for CRC. Fatty liver may promote CRC by increasing insulin resistance, altering the secretion of adipokines, and affecting the balance of inflammatory mediators (22). The association between carotid atherosclerosis and CRC may be related to pathophysiological mechanisms, such as chronic inflammation, oxidative stress, and endothelial dysfunction (6). Notably, this study identified HDL-C as an independent risk factor for CRC, which contradicts traditional views that HDL-C is generally protective against tumor development (23). A retrospective study observed that serum HDL-C levels were significantly increased in CRC patients with ocular metastasis, with levels above 1.27 mmol/L associated with an increased risk of ocular metastasis (24). This threshold was close to 1.615 mmol/L for HDL-C, which was identified as a risk factor for CRC in this study. A Mendelian randomization analysis found higher genetically predicted HDL-C levels were associated with increased risk of non-endometrioid endometrial cancer (25). The findings of this study suggest that HDL-C may not be a universally protective factor for all types of cancer but rather may act as a risk factor for specific tumor types (26), and may also be related to the degree of differentiation or staging of CRC (27). However, the exact causal relationship between HDL-C levels and the development of CRC requires further research to provide definitive evidence. Emerging evidence suggests that the distribution and functional aberrations of HDL-C subtypes may indirectly facilitate cancer progression through alterations in cholesterol metabolism (28). At the genetic level, genome-wide association studies (GWAS) have identified a functional mutation at the rs5888 locus of the SCARB1 gene, which can impede HDL-C metabolism. This leads to the accumulation of oxidized HDL-C in circulation, triggering the release of inflammatory cytokines and subsequently promoting the proliferation of colorectal epithelial cells via the activation of the TLR4/NF-κB signaling pathway (29, 30). These findings highlight that clinical practice should not only focus on HDL-C concentrations but also on its functional status and genetic background, thereby providing novel insights for the early detection and warning of CRC. Additionally, this study highlighted gut microbiota dysbiosis as another risk factor for CRC, emphasizing that an imbalance in the gut microbiota may lead to lipid metabolism disorders. Recent research has shown that an imbalance in the gut microbiota significantly affects lipid metabolism in the host, leading to diseases such as obesity, hyperlipidemia, and NAFLD (31). These findings provide new perspectives for understanding the mechanisms of fatty liver, carotid atherosclerosis, HDL-C, and gut microbiota dysbiosis in CRC and may have significant implications for CRC risk assessment and clinical treatment strategies.

### 4.4 Limitations of this study

Although the FAH model has certain value in CRC prediction, it has limitations. Firstly, there is a lack of external validation. External validation is a crucial step in evaluating the generalization ability of a model, ensuring that the model still exhibits good predictive performance in different populations and environments (32). However, the FAH model is constructed based on specific datasets and lacks validation from independent samples, which raises doubts about its reliability in practical applications. Therefore, future research should focus on conducting multi—center, large—sample external validation to fully evaluate the stability and applicability of the FAH model, thus providing more robust support for the accurate prediction of CRC. Secondly, the FAH model assesses the sensitivity and specificity of the test based on a single screening method. Furthermore, current guidelines identify intestinal polyps as a risk factor for CRC. However, this study found no association between colonic polyps and CRC risk. On the contrary, patients with intestinal polyps exhibited a lower probability of developing CRC. This may be attributed to the surgical removal of polyps following their detection through colonoscopy, thereby placing patients in a disease-free state and reducing the incidence of CRC. This observation indirectly suggests that early detection and intervention in patients with intestinal polyps could potentially decrease CRC incidence. As this was a retrospective study, some patients were excluded, and the analysis may have been biased. Large-sample, multicenter prospective studies are needed to explore the relationship between metabolism-related diseases and CRC.

In conclusion, this study identified NAFLD, carotid atherosclerosis, low HDL-C levels, and intestinal dysbiosis as independent risk factors for CRC. These findings provide a scientific basis for healthcare professionals to implement effective preventive measures, thereby reducing CRC incidence. The FAH model offers a practical, straightforward, and rapid approach for predicting CRC, providing valuable guidance for clinical practice and introducing novel perspectives for CRC screening.

## Data Availability

The raw data supporting the conclusions of this article will be made available by the authors, without undue reservation.
